# Using Contactless Interfacial Rheology to Probe Interfacial Mechanics for Compositional Ripening

**DOI:** 10.1021/acs.langmuir.4c04622

**Published:** 2025-04-30

**Authors:** Raj Tadi, James A. Richards, Fraser H. J. Laidlaw, Beth Green, Thomas Curwen, Andrew B. Schofield, Job H. J. Thijssen, Paul S. Clegg

**Affiliations:** † SUPA School of Physics and Astronomy, University of Edinburgh, Peter Guthrie Tait Road, Edinburgh EH9 3FD, U.K.; ‡ Reading Science Centre, Mondele̅z International, Whiteknights Campus, Pepper Lane, Reading RG6 6LA, U.K.

## Abstract

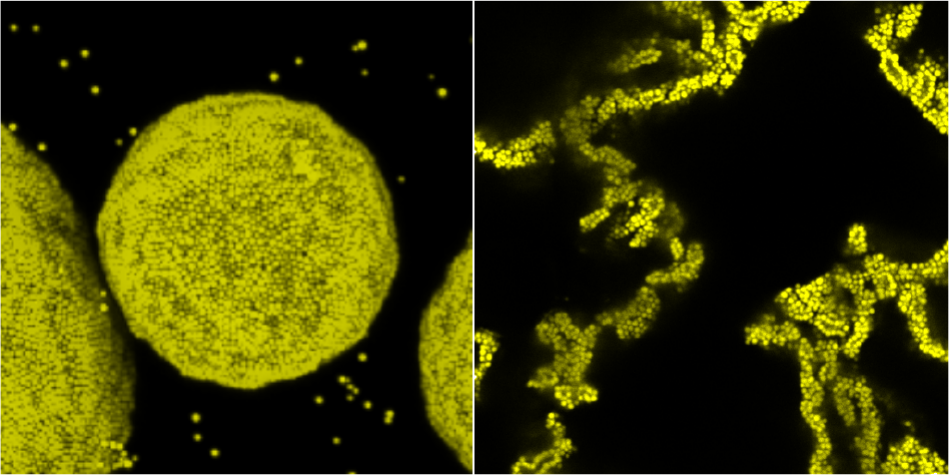

In this study, we investigate the impact of modifying colloid–colloid interactions on the rheological properties of a layer of poly(methyl methacrylate) PMMA colloids at a dodecane-water interface. Toluene is introduced into the oil phase in order to modify attractive interactions between colloids. We first make qualitative observations of water-in-oil emulsions undergoing compositional ripening, demonstrating how the addition of toluene modifies the evolution. Without toluene, water droplets finally “explode”; with the addition of toluene, they instead form connected colloidal structures. We secondly employ a novel contactless interfacial setup to probe the rheological properties of a PMMA colloid-laden water-dodecane interface, examining the effects of toluene addition. We find that the interface becomes significantly weaker and more flexible following addition of toluene, contrary to what one might expect for increasing interparticle attractions for high surface coverage interfaces.

## Introduction

Pickering emulsions are a class of emulsions stabilized by particles. Typically, the particles are in the colloid size domain. Compared to the surfactant-stabilized counterparts, Pickering emulsions show many advantages such as improved stability and tunable properties.^1^ While the stability of emulsions is of great importance to creating long-lived products, understanding the destabilization process is also important for various applications.^2^ For example, in food^3^ and pharmaceutical^4^ applications, where the emulsified species is to be released, and in the separation of Pickering emulsion constituents.^5^ The stability of Pickering emulsions can be attributed to the extremely high colloid desorption energy^6^ making parameters such as the colloid size, three-phase contact angle, and oil–water surface tension, very important. In addition, the rheological properties of the colloid-laden interface have also been shown to strongly correlate with the stability of emulsions.^7^ Naturally, this also places a high importance on how colloids on the interface interact with each other.

Elastic colloidal interfaces can be formed in various ways, ranging from the jamming of hard particles to the use of soft, deformable particles such as microgels.^8^ Various researchers have investigated particle interactions and their effect on the rheological properties of colloidal interfaces. In addition to changes in the surface chemistry of the colloids^9,10^ these are often conducted via the addition of salt, surfactant, or electrolytes to the aqueous phase.^11−13^ At high surface coverage short-range interactions are key.^14^ Generally speaking, the viscoelastic response is thought to be governed by constraints on particle motion^15^ with stronger interactions appearing to show higher viscoelastic parameters.^16^ Work by Rahman et al.^13^ and Barman and Christopher^17^ suggest that the attraction between colloids is the primary determinant of the magnitude of interfacial moduli in shear rheology, with decreasing attraction or increasing repulsion resulting in lower viscoelastic moduli. Yu et al.^12^ showed the transition from viscous-like to solid-like can be reduced via the screening of electrostatic repulsion between particles.

The viscoelastic properties of colloidal interfaces are crucial for the stability of emulsions and foams. In the context of compressional rheology, Rodríguez-Hakim et al.^18^ generalized the criterion for long-term bubble stability to account for slow, unidirectional compression. The significance of shear rheology in bubble dissolution has been highlighted by Beltramo et al.^19^ demonstrating that the emergence of yield stress is essential for countering Ostwald ripening in armored bubbles. They further showed that increased capillary attraction between colloids enhances interfacial yield stress. When droplets or bubbles deform nonuniformly, the deformation dynamics, in addition to interfacial rheology, play a significant role, introducing additional complexities.

In this work, we attempt to alter only the short-range interactions between interfacial PMMA hard-sphere colloids, and thus the interfacial properties. Furthermore, we explore how these changes can affect water-in-oil Pickering emulsions destabilizing via compositional ripening. Instead of a modification via the aqueous phase, here the colloid interactions are altered through the oil phase with the addition of toluene. This is motivated by work by Dinsmore et al.^20^ in which elastic shells of PMMA are formed. The authors suggest toluene destroys the PHSA steric stabilizer, inducing attractive van der Waals interactions, and locking the adsorbed PMMA particles together. By replicating this approach, we expect a greater attraction between colloids, potentially leading to more pronounced viscoelastic properties.

The emulsion work in this paper follows a previous study^21^ in which compositional ripening is utilized as the test of stability for Pickering emulsions. Here a mixed water-in-oil emulsion system is created, stabilized by PMMA colloids, composed of pure water droplets and sugar solution droplets. With a compositional gradient present, mass transfers from the pure water droplets to sugar solution droplets. By varying the oil phases, a difference in emulsion evolution was observed. In the case of dodecane, the water droplets buckle and ultimately “explode” once all the water has been lost, ejecting colloids outward. In contrast, using tributyrin resulted in the water droplets shrinking into tightly compact structures with evidence of water remaining inside. The PMMA dispersions in tributyrin also formed aggregates suggesting changes in the colloid–colloid interactions. Many aspects of the emulsion, such as the three-phase contact angle and interfacial tension, are modified when changing the oil and thus it is difficult to determine the critical parameter behind the observed difference in ripening behavior.

The current work aims to disentangle colloid interactions as a control parameter and to determine whether changes in these interactions alone can affect emulsion stability. To further explore the effects of toluene on the interface, a novel contactless technique^22^ was employed to capture any changes in rheological shear response.

## Results and Discussion

### Emulsion Behavior

#### Pure Emulsions

**Figure 1:**
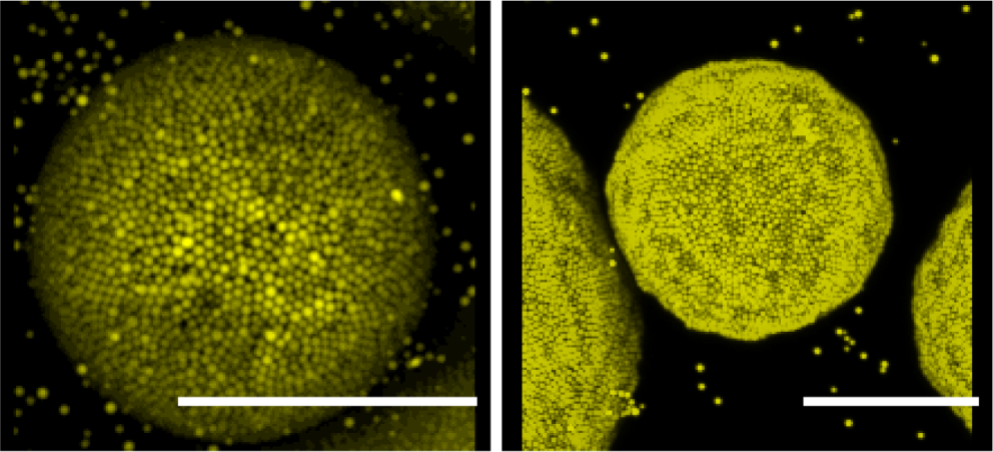
Confocal 3-D projection of a water-in-dodecane droplet stabilized by 360 nm radii PMMA before (left) and after (right) the addition of toluene. Scale bar 20 μm.

We compare water-in-dodecane emulsions stabilized by PMMA particles, which are sterically stabilized by poly(12-hydroxystearic acid) (PHSA), before and after treating the emulsions with toluene. Initially w/o emulsions were created via vortex mixing, likely forming through limited coalescence^23^ as some nonspherical droplets were observed. The toluene treatment involves gently mixing toluene with the emulsion, followed by careful washing to remove it. This process ensures that the droplet structure remains intact and that the continuous phase composition is comparable between treated and untreated emulsions. Further details can be found in the Experimental Section. The resulting toluene-modified emulsions appear similar to those without toluene, with particles forming a dense packed monolayer on the interface, as shown in Figure 1. These emulsions appear stable for over 2 weeks with no clear signs of phase separation. However, the shape of the modified emulsion droplets appears slightly less spherical than the unmodified counterparts, though this may be due to the emulsion being left on a rollerbank for an extended period.

#### Compositional Ripening Comparisons

**Figure 2:**
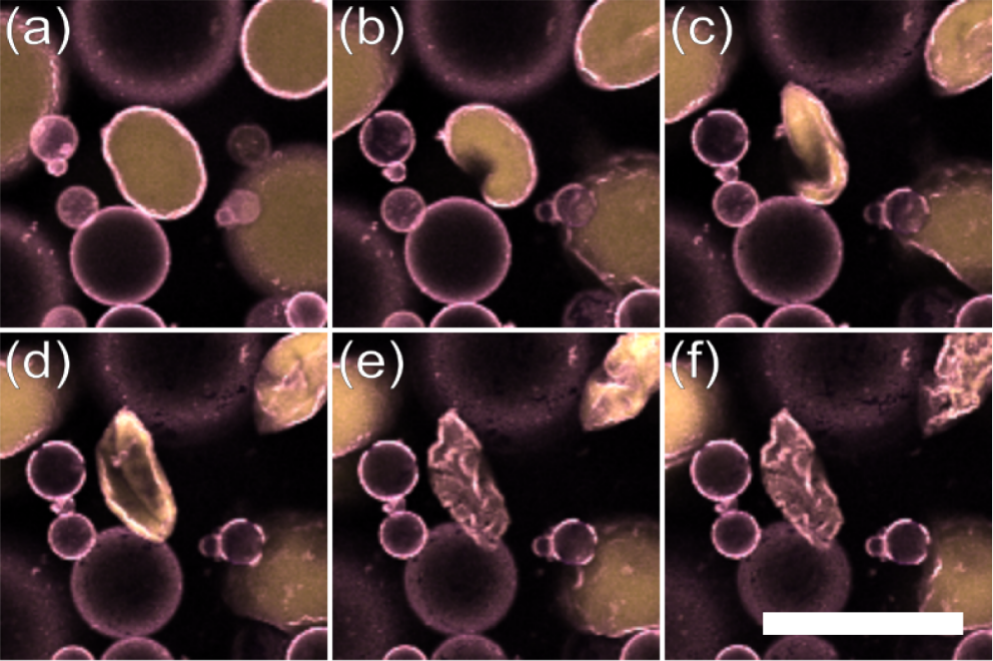
Compositional ripening over 4 h observed at (a) 0 min, (b) 48 min, (c) 96 min, (d) 144 min, (e) 192 min, and (f) 240 min. Toluene-treated water droplets (yellow) stabilized by PMMA (yellow) undergo ripening losing mass to the sugar droplets (dark purple) (a–d). After sufficient mass is lost, the final structure remains the same (e,f). Scale bar 100 μm.

The toluene-treated water-in-dodecane emulsion was then combined with sugar solution-in-dodecane emulsion and observed. The sugar solution-in-dodecane emulsions were not treated with toluene in order to isolate any changes to only the water droplets. Similar to the untreated water-in-dodecane emulsion system^21^ water appears to migrate from the pure water droplets to the sugar-filled ones. This is confirmed by the crumpling of the water droplets, in which their interfaces compress and buckle, and by the sugar droplets growing and coalescing, Figure 2a–c. Interestingly, the end fate of this system does contrast those observed for the nontoluene system. For the toluene-treated droplets, droplet sizes eventually plateau and only partially fragment, Figure 2d–f.

**Figure 3:**
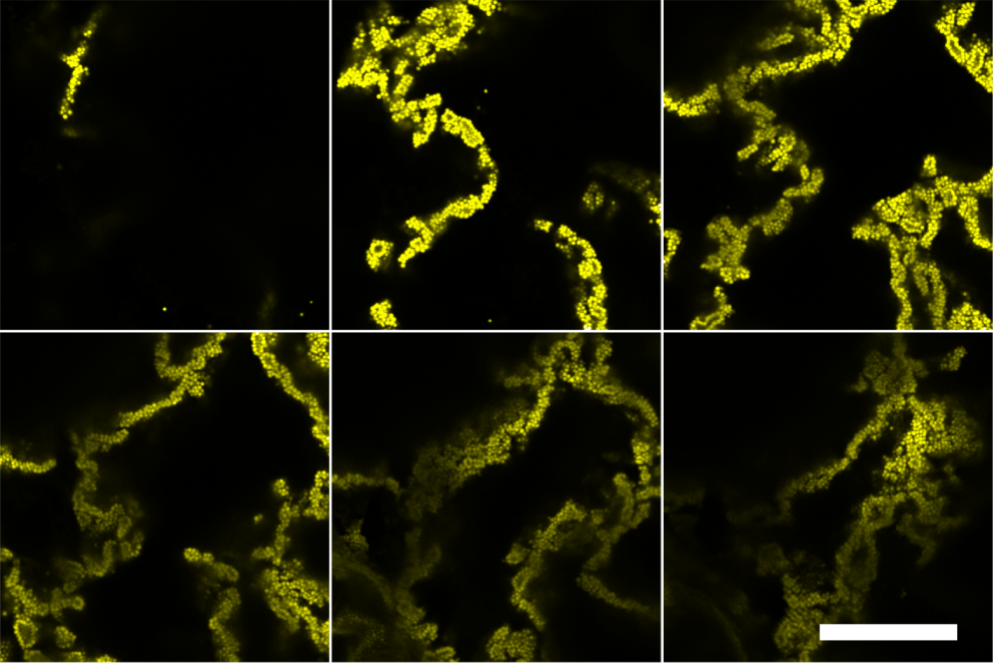
Confocal slices through a toluene-treated water droplet remnant, acquired at 4.5 μm intervals, after 15 h of compositional ripening, showing connected PMMA structures (yellow). Scale bar 25 μm.

Upon closer inspection, the relics of water droplets appear to be filaments or sheets of colloids devoid of water. This is illustrated in Figure 3, where taking a z-stack of confocal images through the sample shows structures that extend several tens of microns instead of a sedimented layer of colloids. This unique end fate for the water droplets may be considered as an intermediate between the dodecane and tributyrin systems explored previously^21^ where droplets neither “explode” nor crumple into very compact structures. This result suggests that there is indeed a change in colloidal interactions through the addition of toluene, although not one in which compositional ripening is overcome. However, the ripening process may be potentially slower in the toluene-treated scenario, as evidenced by the observations of some water droplets remaining after 10 h. Although, without accounting for emulsion characteristics, such as droplet radii and variations in local environment, this cannot be confirmed.

**Figure 4:**
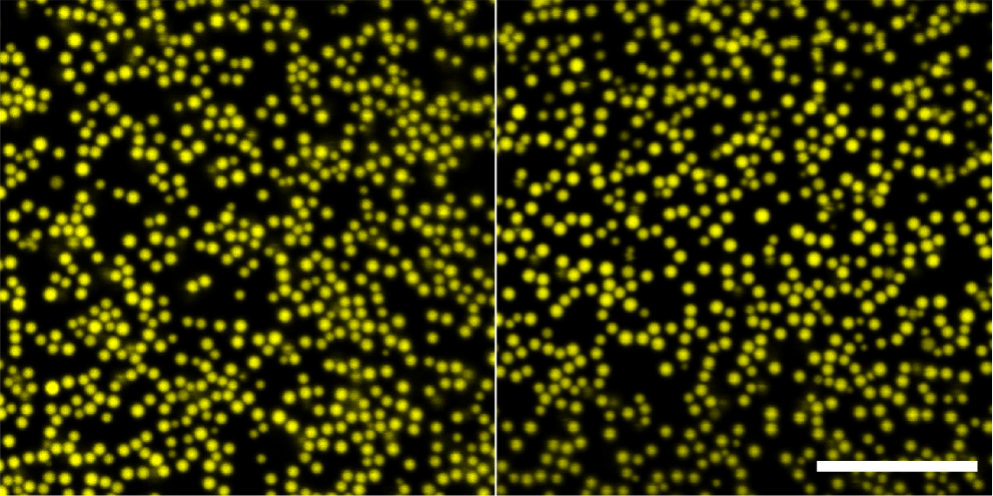
Confocal micrographs of a PMMA dispersion in pure dodecane (left) and after rollerbank overnight in 50:50 dodecane/toluene mixture (right). In the bulk oil phase the behavior of these colloids do not appear to be significantly altered via the addition of toluene. Scale bar 10 μm.

To further investigate any changes in colloid interactions that may be taking place and drive the differences in response to compositional ripening, the PMMA dispersions were explored. Comparing PMMA colloidal dispersions in dodecane with and without the addition of toluene, showed that the particle interactions in bulk were not strongly modified, with colloids showing no difference in aggregation, illustrated in Figure 4. This perhaps brings into question the type of interactions that are being modified for the colloids. Furthermore, emulsions were investigated with Cryo-SEM, where it was observed that upon the addition of toluene, emulsions are not greatly altered (Figure S1). Qualitative observations of contact angles and colloid shape show no large change. Upon measurement, colloid sizes remain unchanged with similar interquartile ranges (Figure S2), suggesting that any changes in the particle swelling cannot be observed on these scales.

### Interfacial Shear Rheology

Having observed two different end fates with and without the addition of toluene, and ruling out changes in the size, contact angle, and attraction in bulk phase, we use interfacial shear rheology to investigate the effects of toluene on short-range interactions between particles. To understand the role of particle interactions through to mechanical failure requires accessing a broad range of interfacial stresses. While the low stress linear-viscoelastic region is readily accessible to magnetic needle interfacial shear rheometry^24^ and the high-stress region to the double-wall ring geometry on a conventional rotational rheometer^25,26^ to access both regimes with the same technique we utilize a novel contactless interfacial shear rheology technique.^22^ While conventional techniques directly attach to the interface, the contactless approach involves shearing the liquid phase above the interface with a rheometer and parallel plate geometry while simultaneously imaging the interfacial response, detailed in Experimental Section. This fluid flow in the upper oil phase transmits stress to the interface. When the interface resists shear deformation, flow in the lower aqueous phase is negligible and the total stress is borne by the interface. The interfacial stress, σ^*s*^, for this setup can be described by
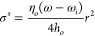
1where ω is the fixed angular velocity of the geometry, ω_*i*_ is the interfacial angular velocity, *r*, is the radius at which the measurement is being made (in this case 5 mm), *h*_*o*_ is the oil phase depth, and η_*o*_ is the oil phase viscosity. When using this technique one should be mindful of applicability regime following eq 1. For the contactless setup a modified Boussinesq number, Bo* is used and described as the following:
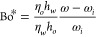
2where *h*_*w*_ and η_*w*_ correspond to the water height and viscosity, respectively. Similar to the classic Boussinesq number, (Bo), used in methods such as the DWR, this should be maximized to ensure the surface contribution is far greater than the bulk phase. In this work, it is observed that ω_*i*_ ≪ ω thus achieving sufficiently high Bo*. From this σ_*s*_ is calculated using ω_*i*_ = 0.

**Figure 5:**
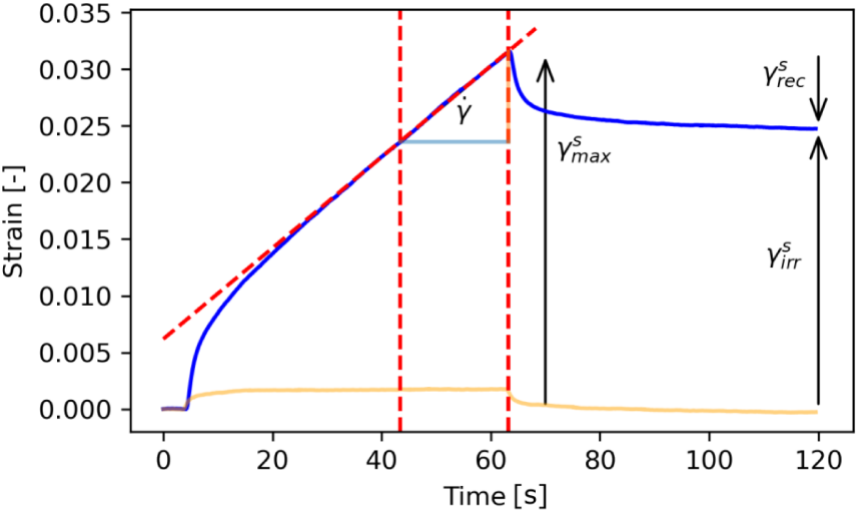
A Creep-recovery profile for an interface showing flowing behavior (blue) reaching a steady strain rate, γ̇. Illustrated are the maximum, γ_max_, recoverable, γ_rec_, and irrrecoverable, γ_irr_ strains. The strain rate, γ̇, is determined by taking the gradient of the steady strain toward the end of the applied stress, over a 20s window (red dotted lines). Also shown is the creep-recovery curve for an interface showing very elastic behavior (orange), illustrating considerably lower values.

A series of creep recovery tests were performed, in which stress is applied to the interface via shearing of the oil phase at fixed rotation rates, ω, while simultaneously imaging fluorescent tracer particles on the interface. The protocol consists of measuring the interface under fixed shear for 60 s followed by 60 s of zero shear. By imaging the tracer particles, a displacement of the interface, and thus a strain, can be determined. From the creep recovery profiles, several strain values were extracted to characterize the interface, Figure 5. The maximum strain, γ_max_, is obtained at the end of the applied stress. The irrecoverable strain, γ_irr_, we define as the total change in strain from the start of rotation to after recovery and indicates the inelastic/plastic behavior of the interface. The recoverable strain, γ_rec_, we define as the change in strain from the peak to the end of recovery and indicates the elastic behavior. Furthermore, the strain rate, *γ̇*, can be approximated when the interface is undergoing steady strain.

By taking a series of creep recovery tests with various stresses, viscoelastic parameters can be measured. During creep tests, a viscous material will initially deform and then reach a steady rate of straining^27^ and the interfacial shear viscosity, η^*s*^, can be determined as η^*s*^ = σ^*s*^/*γ̇*. For an elastic material, an effective shear modulus, *G*_eff_, can be approximated via the Hookean relationship, σ^*s*^ = *G*γ. We determine this from the gradient of the σ^*s*^ and γ_rec_ graph, while the interfaces are behaving predominantly elastically i.e., below any observing flowing behavior γ_rec_ ≫ γ_irr_. Furthermore, the interface exhibits a critical stress above which it readily flows, thus we characterize the interface as having a yield stress, . Defining this precisely is difficult due to the finite time frame of practical creep-recovery tests^28^ and thus we consider two aspects. First, where η^*s*^ sharply drops from the steady state behavior. Second, the transient strain behavior, specifically, we take σ_*y*_ as when γ_irr_ begins to dominate over γ_rec_, indicated by a large increase in γ_max_ and diminishing values of γ_rec_/γ_max_.

#### Without Toluene

**Figure 6:**
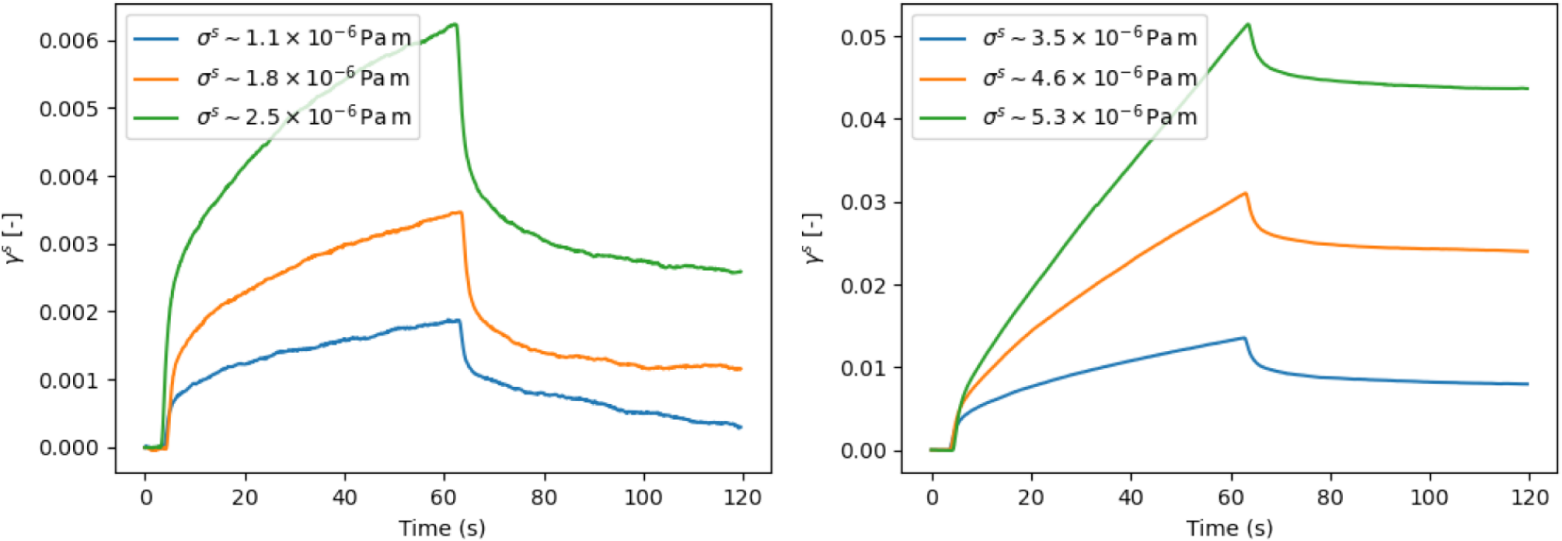
Effect of increasing the stress, via the rotational speed, ω, on the interfacial creep recovery profile. Left: the interface shows a fairly elastic response with a high recoverable strain (ω = 3 rpm; 1.1 × 10^–6^ Pa m) and begins to show plastic behavior as the stress is increased . Right: the interfaces become dominated by plastic strain and flow with a clear constant strain rate for ω higher than 10 rpm; 3.5 × 10^–6^ Pa m. See main text for details.

Initially a PMMA colloid-laden interface with no toluene was prepared and investigated, described in the Experimental Section. Given the colloid interface is well compressed in the emulsion observations, interfaces with close to maximum packing fractions were prepared. As single particle resolution is not possible exact packing fractions cannot be determined. Only interfaces that were homogeneous (Figure S3) were analyzed. Creep recovery experiments were performed with increasing interfacial stress. This is illustrated in Figure 6, where the geometry rotation speed, ω, is incrementally increased from 3 to 15 rpm, corresponding to incremental increases in interfacial stress, and the creep-recovery response measured. At low interfacial stresses, there is an initial jump in the strain which slows down with time indicating a high interfacial viscosity. Additionally γ_rec_ is larger than γ_irr_, indicative of elastic behavior. As the stress is increased, the initial deformation increases while the interface appears to decrease its elastic recovery, indicating the beginning of plastic flow. At high stresses, the interface appears to be flowing, with a clear linear relationship between strain and time being achieved. Alongside, a considerably lower recoverable strain compared to irrecoverable strain is seen, which suggests the interface is undergoing a strong plastic deformation. This yielding behavior is also observed for the high surface coverage PMMA oil–water interface measured by Muntz et al.^22^ The authors also demonstrated an irreversible effect of shear for PMMA colloid dodecane-water interfaces. To determine the experimental design for the addition of toluene, we also explore this aspect.

**Figure 7:**
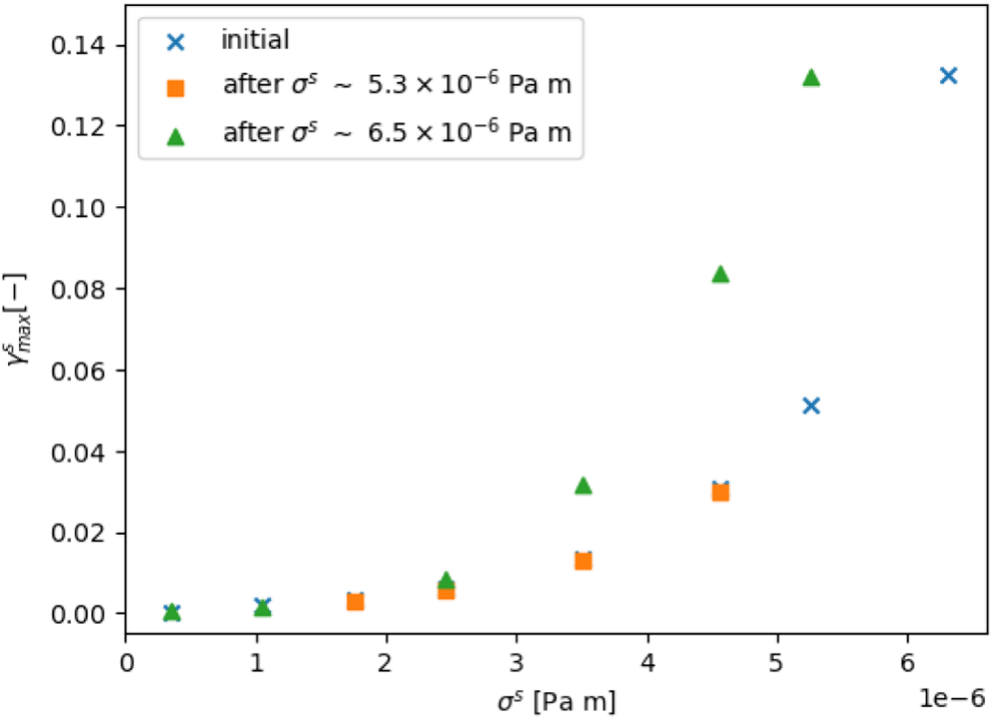
Effects of shear history on the interfacial shear response on a PMMA (362 nm radius) water-dodecane interface without toluene. The crosses indicate the initial response when the interfacial stress, σ^*s*^, is increased. The orange squares are measurements made after σ^*s*^ = 5.3 × 10^–6^ Pa m. The triangles are measurements made after increasing to σ^s^ = 6.5 × 10^–6^ Pa m.

Hysteresis loops consisting of incrementally increasing the applied stress to certain values, then incrementally decreasing it back down to observe any changes in the creep recovery behavior were performed, illustrated in Figure 7. Any significant differences would be indicative of irreversible changes to the interface upon shear. Initially ω was incrementally increased to 15 rpm (blue crosses in Figure 7 up to σ^*s*^ ∼ 5.3 × 10^–6^ Pa m), where the interface was starting to flow, then incrementally decreased (orange squares in Figure 7). In this situation, preshearing the interface to 15 rpm resulted in no significant difference in the interfacial strain response, with the maximum strain curves lying close to each other. Next, ω was set to 18 rpm (blue cross in Figure 7 around σ^*s*^ ∼ 6.5 × 10^–6^ Pa m), where the interface was well into the plastic regime, indicated by the large jump in maximum strain. The applied stress was then decreased incrementally (green triangles in Figure 7). In this case, there is a drastic difference with a divergence of the two curves. Compared to the nonsheared interface, the 18 rpm presheared interface exhibits higher maximum strains, particularly under stresses significantly exceeding the yield stress of approximately 3 × 10^–6^ Pa m. For example, at σ^*s*^ = 5.26 × 10^–6^ Pa m, γ_max_ increases from 0.0436 to 0.123. This suggests a weakening of the interface induced by preshearing at high enough stresses, specifically 18 rpm here.

Looking at how the effective elastic modulus, *G*_eff_, changes under increasing maximum stress, we initially find this to be 6.54 × 10^–4^ Pa m, upon preshearing to 15 and 18 rpm this decreases to 6.35 × 10^–4^ Pa m and 5.57 × 10^–4^ Pa m, respectively. From the data the yield stress,  is indicated by the large increase in interfacial strain ≈ 3 × 10^–6^ Pa m. These viscoelastic parameters are in approximate agreement with those obtained by Van Hooghten et al.^29^ where the authors report G ∼ 6.1 × 10^–4^ Pa m, and  ∼ 5 × 10^–6^ Pa m, for near maximally packed 0.455 μm radius PMMA at a water-hexadecane interface. Our interfacial rheology results so far, without toluene treatment, compare relatively well to literature, which provides confidence for the comparative measurements following toluene treatment.

#### With Toluene

**Figure 8:**
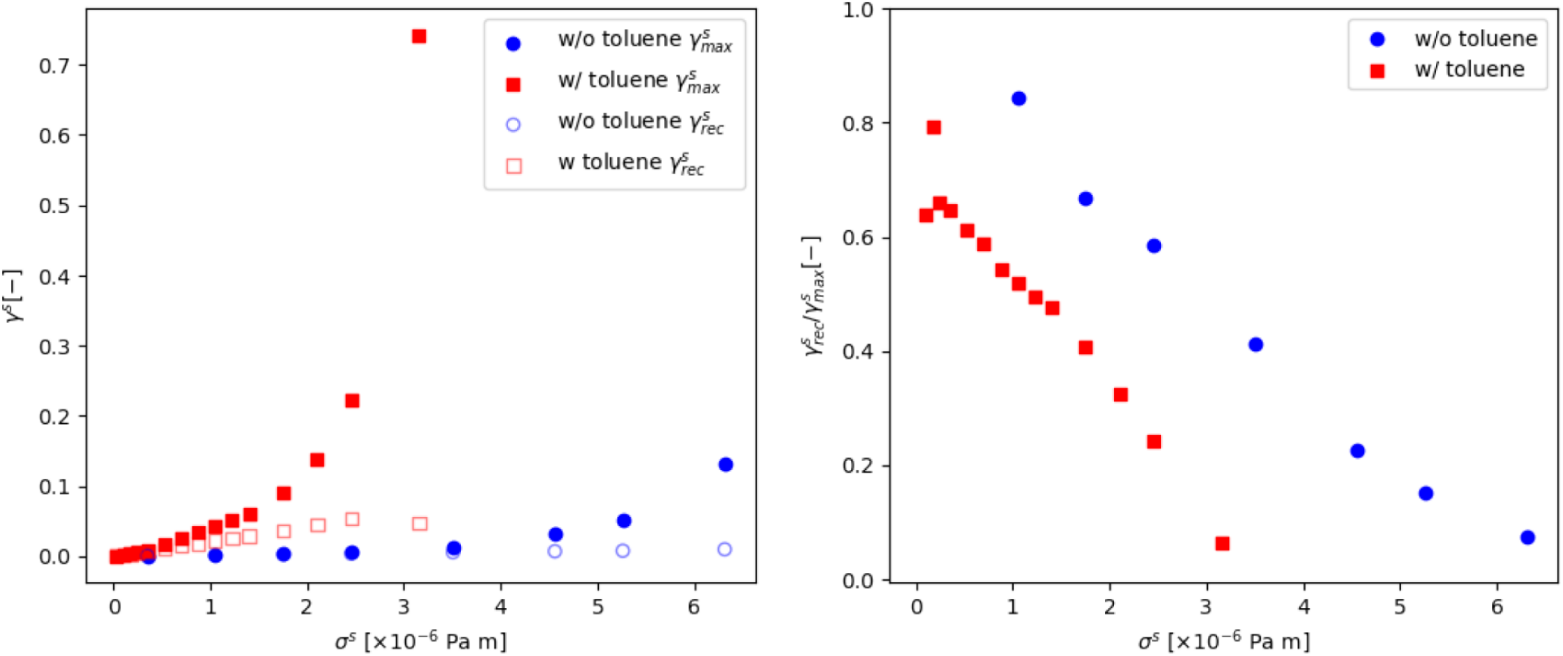
Left: the maximum,  (closed), and recoverable, (open), strains as a function of interfacial stress. Right: The ratio of / before (blue circles) and after (red square) the addition of toluene to the interface.

With it now understood that the interface exhibits some shear history dependence, where preshearing at a certain stress can significantly weaken the interface, the effects of toluene were explored on this interface. Furthermore, this approach mirrors the emulsion preparation process, which involves mixing followed by the addition of toluene. Toluene was added to the presheared interface, and creep recovery tests were performed with incremental levels of applied stress, similar to before. The maximum, , and recoverable, , strains were extracted from the creep-recovery curves, Figure 8. The changes are very pronounced, with the toluene-modified interfaces showing a much greater strain response for a given stress, compared to the nontoluene interface (blue circles), indicative of a weaker interface. For example, at σ^*s*^ = 2.5 × 10^–6^ Pa m, γ_max_ increases from 6.2 × 10^–3^ to 2.2 × 10^–1^. Looking at  and  also provides a more quantitative indication of changes in the yield stress. Where  increases more than Figure 8 (left) and  becomes to dominate, we define  by / ∼ 0.5 Figure 8 (right). Upon the addition of toluene, this operative yield stress decreases noticeably, from around 3 × 10^–6^ Pa m to 1 × 10^–6^ Pa m.

Measurements of elastic moduli before and after the addition of toluene also show a significant change. For the unmodified interface *G*_eff_ ∼ 5.57 × 10^–4^ Pa m, whereas upon toluene addition *G*_eff_ ∼ 5.12 × 10^–5^ Pa m, an order of magnitude in difference.

**Figure 9:**
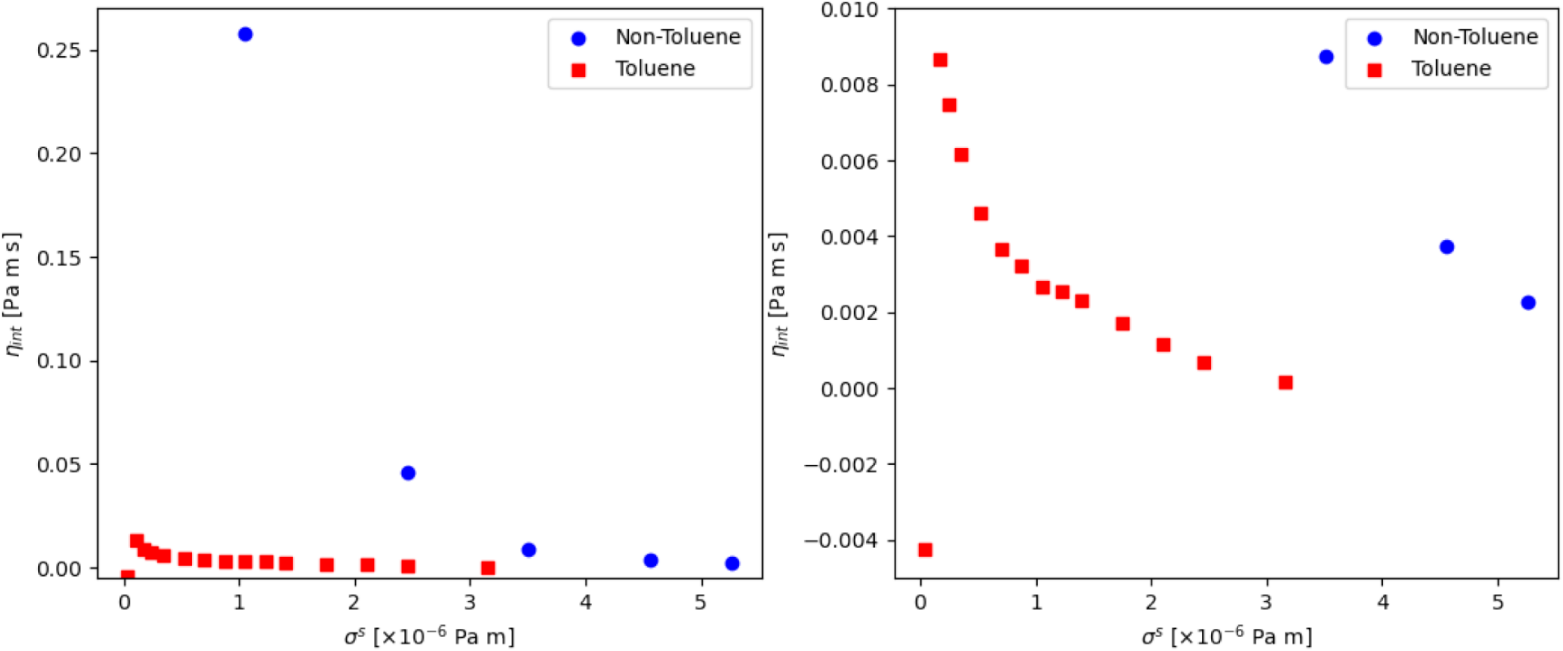
Left: The effects of increasing stress on the interfacial viscosity before (blue circles) and after (red squares) the addition of toluene. Right: A closer look at the viscosity-stress curve, where the variation in the toluene data is more evident.

The interfacial viscosity, η^*s*^, is also assessed, Figure 9. The difference in viscosities is very large, illustrative of a much weaker interface when toluene is added. Additionally, a yield stress is more obvious in these plots, indicated by the “knee” in the η^*s*^ - σ^*s*^ curves, in line with the yield stress determined from  and , shown in Figure 8.

**Figure 10:**
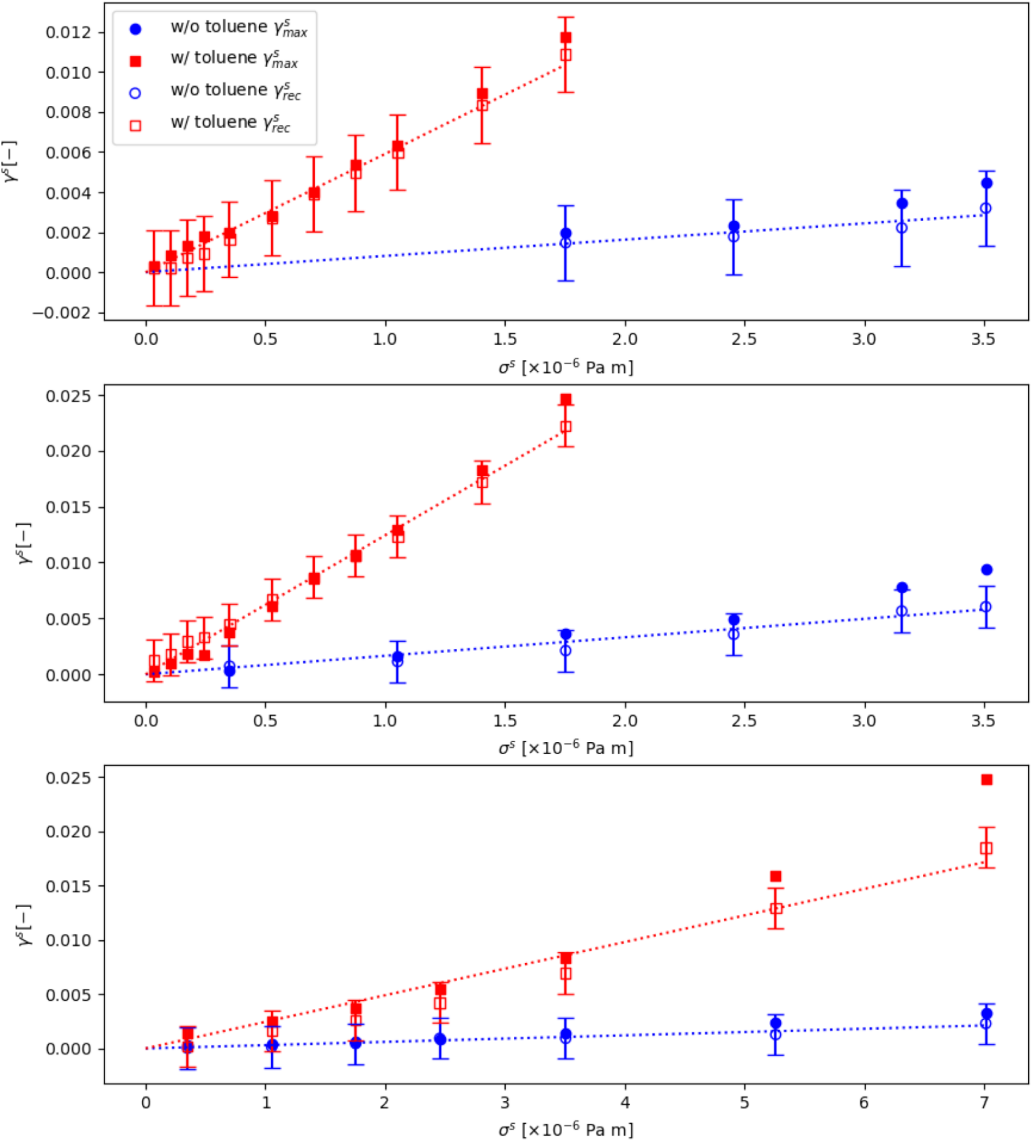
Maximum (closed) and recoverable (open) strains as a function of interfacial stress before (blue circles) and after (red squares) the addition of toluene to the interface for 3 different runs (top to bottom). Straight lines are also fitted to the recoverable strain to derive an “effective” shear modulus.

The decrease in rheological properties, *G*_eff_, η^*s*^, and  contrasts with what was expected for the case of increasing particle–particle attractions of a maximally packed interface.^17^ From these results, it had to be determined whether the stark change in rheological behavior was only due to toluene modifying interactions, or whether preshearing the interface had a substantial role to play. As a result, three interfaces were prepared and creep recovery runs of incremental stresses were performed on the interfaces until there were signs of the interface beginning to flow, being careful to remain below the threshold for irreversible structural changes. Toluene was then added and creep recovery tests performed again. While the interfaces were prepared for maximum packing, particle level control was not possible which naturally leads to uncertainties in the area fraction of particles. As a result, interfaces showed variance in their properties. The stress–strain graphs, showing both γ_rec_ and γ_max_, for the three separate interfaces are illustrated in Figure 10. In all three runs, the effect of added toluene is clear: a large reduction in effective shear modulus and thus a much weaker interface.

The values for *G*_eff_ before and after the addition of toluene are summarized in Table 1. Before the addition of toluene, interfaces are comparable to those reported by Van Hooghten et al.^29^ with G ∼ 6 × 10^–4^ Pa m, for a maximally packed interface. The deviations in our values we attribute to difficulty in preparing identical interfaces, where small changes in surface coverage can have large effects on the interface.^14^ Interestingly, even though the interfaces vary in *G*_eff_ from 3.31 × 10^–3^ Pa m to 6.04 × 10^–4^ Pa m, the addition of toluene consistently decreases the modulus by roughly a factor of 7.5. This confirms the role of toluene in weakening the interface rather than weakening due to structural changes from preshearing the interface. Such changes may also impact the viscosity, however given we are operating in the elastic regime, where noise and drift can become quite prominent, accurate comparisons for the interfacial viscosity cannot be made.

**Table 1:** Effective Shear Moduli, *G*_eff_^s^, Before and After the Addition of Toluene to the PMMA Water-Dodecane Interface

Run, preshear [Pa m]	*G*_eff_^s^ pre-toluene [Pa m]	*G*_eff_^s^ post-toluene [Pa m]	Factor
1, -	6.54 × 10^–4^	-	-
1, ∼5.3 × 10^–6^	6.35 × 10^–4^	-	-
1, ∼6.5 × 10^–6^	5.57 × 10^–4^	5.12 × 10^–5^	10.88
2, -	1.23 × 10^–3^	1.69 × 10^–4^	7.27
3, -	6.04 × 10^–4^	8.04 × 10^–5^	7.51
4, -	3.31 × 10^–3^	4.08 × 10^–4^	8.11

#### Discussion

Bringing all the above results together, the following picture emerges. Toluene is added to water-in-dodecane emulsions stabilized by PMMA colloids, which are then subject to compositional ripening via the addition of sugar-in-dodecane PMMA-stabilized emulsions. The evolution of these water droplets, as they lose mass appears similar to those without toluene treatment. In both cases, water droplets shrink and their interfaces buckle, with no significant difference in their ripening rates. However, the contrasting behavior comes in their end fate. Water droplets without toluene treatment eventually “explode”, whereas droplets treated with toluene form connected colloid structures devoid of water. This suggests interactions have been modified such that they “stick” together instead of being ejected outward. However, the changes in interfacial properties are insufficient to overcome the ripening process. Furthermore, the behavior of the colloids in bulk dodecane with toluene did not appear to change substantially, bringing into question whether greater van der Waals interactions were being induced.

Shear interfacial rheology was explored as a means of understanding what properties of the interface were being modified by toluene via the use of a contactless rheology setup. Interfaces with high colloid surface fractions were prepared, mimicking the interface of emulsion droplets, and were shown to behave like a yield stress material, similar to the current literature.^22^ When toluene was added, this appeared to significantly and progressively decrease the elastic modulus, consistent with generating a much weaker interface. There was also some indication that the yield stress and interfacial viscosity decreased upon the addition of toluene. While it has been demonstrated in the literature that aggregation of colloids on interfaces can lead to weaker interfaces due to “voids” forming,^22,29,30^ in these scenarios the packing fraction is below the maximum. For interfaces with higher surface coverages aggregation can lead to higher interfacial viscosities.^17^ Although in our study it is difficult to ascertain an exact surface coverage as single colloid resolution is not possible, given that the viscoelastic properties are comparable to that of a similar interface with close-packed colloids^29^ this suggests we indeed have high packed surface coverage. Furthermore, the cryo-SEM micrographs do not show clear signs of voids. These results provide further evidence that the changes in interparticle interactions may not originate from the destruction of steric stabilization as initially thought.

The observed differences in emulsion outcomes could be explained from the perspective of brittle to ductile behavior. The interface without toluene is somewhat brittle in which there is little plastic deformation before fracture. Whereas with toluene this behaves more ductile, where a greater plastic deformation is attained before fracture. However, it is important to note that clear indicators such as a stress drop or shear localization^31^ are inaccessible for our particular protocol. A potential reason for the decrease in viscoelastic properties may be toluene changing the physical structure of the colloids. Toluene is considered a good solvent for PMMA^32^ and it may be the case that it has a plasticizing effect that creates a soft layer only a few nanometers thick, similar to a system of PVC colloids and Dinch solvent.^33^ If this is the case, one might expect solvents such as methyl methacrylate or acetone to have similar effects. The softening of colloids, at fixed surface coverages, therefore could allow more freedom in their motion and thus lower viscoelastic properties, analogous to 3D bulk behavior.^34^

## Experimental Section

### Materials

Poly(methyl methacrylate) (PMMA) colloids sterically stabilized with poly(12-hydroxystearic acid) (PHSA) hairs were used, dyed with NBD (*r* ≈ 362 nm) or Nile red (*r* ≈ 1887 nm), or left undyed (*r* ≈ 355 nm). The radii were measured with dynamic light scattering (DLS) in dodecane. The aqueous phases used were water (deionized using a Milli-Q reagent system with resistivity of 18 MΩ cm) and a sugar solution (at 64% weight fraction) made from dissolving consumer table sugar (Sainsburys) in deionized water. The sugar solution was dyed with Rhodamine B. The oils used were n-dodecane (Sigma-Aldrich 99%) and toluene (Sigma-Aldrich 99%).

### Emulsion Preparation and Characterization

To prepare the continuous phase, dry PMMA particles were dispersed in dodecane at a volume fraction of 1% using a pulsed sonicator probe (Sonics Vibracell). The protocol consisted of a 5 s sonication followed by a 5 s rest, repeated for a total time of 40 min, at an amplitude of 20%. This ensured the colloids were well dispersed with no aggregates.

Water-in-oil and sugar solution-in-oil emulsions were all prepared at 20% volume fractions, using 1 g of the 1% PMMA dodecane dispersion. The aqueous phases were added to the dodecane PMMA dispersion and vortex mixed, until the oil layer became clear, confirming that essentially all the PMMA had adsorbed onto the water–oil interface.

For the toluene treatment, a solution of dodecane at 50% toluene volume fraction was first prepared. This solution, typically 1 g, was then added to the water-in-dodecane PMMA emulsion and left on a rollerbank overnight to gently mix with the colloids. The emulsions were then washed with dodecane to remove excess toluene, via the following protocol: Adding 4 g of dodecane, gentle rollerbank for 15 min, allowing the emulsion to sediment for 10 min, then carefully siphoning 4 g of the continuous phase. This was repeated 5 times, and for the final repeat, an extra 1 g was removed to return the emulsion to a roughly 20% volume fraction.

Equal volumes of the water-in-dodecane and sugar solution-in-dodecane emulsions were combined, shaken gently for a few seconds then pipetted onto a cavity slide and immediately observed with a Zeiss LSM 700 confocal microscope. A 488 nm laser was used to observe the sodium fluorescein and NBD dyed colloids, and a 555 nm laser for Rhodamine B.

### Interfacial Rheology Preparation and Protocol

The interfacial rheology consists of a Leica SP8 confocal microscope using a ×10 objective and an Anton Paar MCR 301 stress-controlled rheometer using a 25 mm parallel plate geometry. A spreading solvent mixture is first prepared, consisting of the small PMMA colloids to be investigated (dyed with NBD, *r* ≈ 362 nm) as well as larger PMMA tracer particles (dyed with Nile Red *r* ≈ 1887 nm). This was prepared by first dispersing the small and large PMMA colloids in hexane to produce mass fractions of 1% and  % respectively, this was then diluted with isopropanol to reduce the volume fractions to 0.5% and  % respectively. These mass fractions were specifically chosen such that the interfacial coverage is roughly 90%, close to the maximum packing fraction, with the larger tracer colloids covering only 0.1% of the colloidal surface. Interfaces were prepared using a polytetrafluoroethylene (PTFE) cup with an aluminum ring insert, similar to that of Muntz et al.^22^ The cup was filled with water to a height of 3 mm, ensuring it was pinned to the edge of the aluminum ring, and then 50 μL of the spreading solvent was carefully pipetted onto the interface. Following this dodecane was carefully pipetted to a height of ≈ 2.5 mm (≈3.7 g). The parallel-plate geometry is then contacted to the oil-air interface ensuring it is centered, the microscope stage is then adjusted such that the field of view is at a radius of 5 mm. Using the 488 nm laser, exciting the smaller NBD-dyed colloids, the interface coverage can be observed, ensuring a homogeneous interfacial coverage. The 552 nm laser excites the larger Nile Red dyed tracer particles, used for the particle image velocimetry (PIV) algorithm and ultimately observing the shear response. Velocimetry of the tracer particle was performed using the OpenPIV^35^ python package. This calculated a displacement of the tracer particles between frames, providing a net displacement when summed over all frames. This displacement is then converted to a strain by dividing by the distance from the measurement to the outer wall, in this case, 5 mm. Creep recovery protocols^36^ consisted of a fixed rotation rate for 60 s, followed by zero rotation rate for 60 s, while simultaneously imaging the interface at 25 fps. This results in a video of 512 × 512 pixels (931 μm × 931 μm) and 3000 frames. Performing PIV on the resulting video then yields a creep recovery strain profile. To validate the PIV approach, the maximum and final displacements were measured by hand in ImageJ. The PIV parameters were optimized such that the creep recovery profiles fell within one standard deviation of by-hand measurements.

To investigate the effect of toluene on the interface, a 50:50 toluene/dodecane mass fraction solution was first prepared. Approximately 1.4 g of this solution was then pipetted into the continuous phase, being careful not to disturb the interface. This was then left for 20 min to mix with the remaining continuous phase. After this ≈1.4 g of the oil phase was removed, to return the oil phase to a height of ≈2.5 mm such that the geometry was pinned to the oil-air interface again.

## Conclusions

In conclusion, we have investigated the modification of short-range interactions between PMMA colloids on the water-dodecane interface by altering the oil phase through the addition of toluene. We examined how these changes affect the behavior of PMMA-stabilized water-in-oil emulsions during compositional ripening. In both cases, water droplets deform by buckling; however, once modified by toluene, the colloids remain as intact structures rather than eventually being ejected outward (“exploding”). Contactless shear rheology confirms that toluene consistently weakens the interface, which implies that the more ductile interface prevents the particles from being ejected outward. We speculate that this may be due to colloids becoming softer, instead of short-range particle–particle interactions being modified. Overall, this work highlights the critical role of interfacial rheological properties in determining emulsion behavior and potentially opens routes for tailoring emulsion stability and deformation dynamics.

## Supporting Information Available

Cryo-SEM characterization of water-in-dodecane emulsions without, with, and after washing toluene, and confocal micrographs of the interfacial rheology setup (PDF)
