# Supplementary material for: Using Contactless Interfacial Rheology to Probe Interfacial Mechanics for Compositional Ripening
Source: Langmuir. 2025 Apr 30;41(18):11339–48. doi: 10.1021/acs.langmuir.4c04622 (PMC12080336; doi:10.1021/acs.langmuir.4c04622)
Supplement: Supplementary file 1 — la4c04622_si_001.pdf [file la4c04622_si_001.pdf]

Supporting Information

Using contactless interfacial rheology to probe interfacial mechanics for compositional ripening

Raj Tadi,[*,†] James A. Richards,[†] Fraser H. J. Laidlaw,[†] Beth Green,[‡] Thomas Curwen,[‡] Andrew B. Schofield,[†] Job H. J. Thijssen,[†] and Paul S. Clegg[†]

†*SUPA School of Physics and Astronomy, University of Edinburgh, Peter Guthrie Tait Road, Edinburgh EH9 3FD, UK*

‡*Mondelēz International, Reading Science Centre, Whiteknights Campus, Pepper Lane, Reading RG6 6LA, UK*

E-mail: raj.tadi@ed.ac.uk

Keywords: interfacial rheology, pickering, emulsions, compositional ripening, colloids

Number of pages: 4

Number of figures: 3

Number of schemes: 0

Number of tables: 0

Table of Contents

1. Emulsion characterisation using Cryo-SEM S2

2. Confocal microscopy of the interfacial rheology setup S2

Cryo-SEM characterisation

Emulsions were also characterised using cryo-scanning electron microscopy (Cryo-SEM). Cryo-SEM was carried out on a Zeiss Crossbeam 550 fitted with a Quorum Technologies PP3010T. Samples were prepared by filling glued copper rivets and freezing in nitrogen slush before mounting the rivet in a cryo stub under liquid nitrogen. Fracture of the sample was done by pushing the top of the rivet off in the vacuum of the Quorum PP3010T preparation chamber. The interfaces were sublimed for 5 min $-90°$C, followed by sputtering a conductive Pt layer. Images were taken with the stage at $-140°$C and with an accelerating voltage of 1 kV and a beam current of 100 pA using the secondary electron and Inlens detectors.

Cryo-SEM images of the water-in-dodecane emulsion before toluene is added, after toluene is added, and after cleaning, are provided in Fig. S1. To highlight the various phases these are falsely coloured colloids (green), oil (red), and water (blue). Freeze fracturing sometimes ensures cross cross-sections of droplets can observed, which allows some insight into the size of the colloids and their contact angles with the oil-water interfaces. The images show that the colloids remain very hydrophobic in all three emulsions. Furthermore, the colloid radii were measured and summarised in Fig. S2. The number of colloids measured for the unmodified water-dodecane emulsions, those with the addition of toluene, and after washing the emulsion, were 47, 44, and 45 counts respectively. Here there appears to be no significant difference in colloid size across the three samples, with similar median values and interquartile ranges.

Interfacial Rheology Microscopy

Confocal micrographs of the interfacial rheology setup are provided in Figure. S3. Only interfaces with a homogenous layer of PMMA are chosen to be analysed Fig. S3 (left), as these reflect the coverage on the emulsion droplets. It is worth noting in this setup it is not possible to resolve single colloids of this size (362nm). For the PIV algorithm the larger tracer particles are excited, which can be individually resolved Fig. S3 (middle) and their motion tracked. Interfaces which appeared to be highly inhomogenous with voids and islands

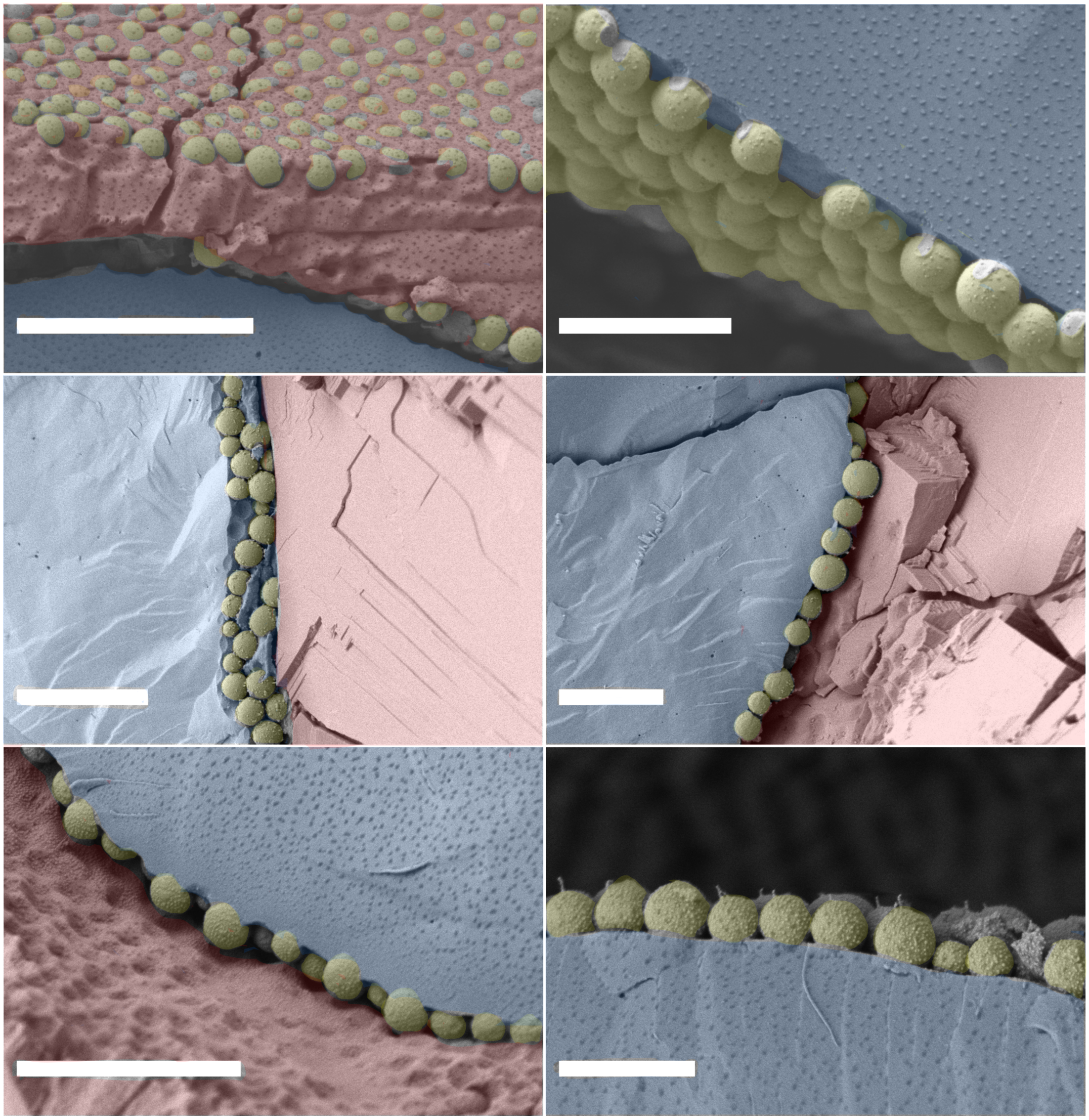

Figure S1: Freeze fracture cryo-SEM images of water-in-dodecane emulsions stabilised by PMMA. Without toluene (top), after adding toluene (middle), and after washing the emulsion (bottom). The various phases are falsely coloured: colloids (green), oil (red), water (blue). The colloids have quite high contact angles, and thus are expected to mostly sit in the oil phase when attached at an interface. This is evident when looking at the cross sections of water droplets as shown in the images. The small specks are likely frost formation. Scale bars of 3 μm.

of particles were not used for analysis Fig. S3 (right).

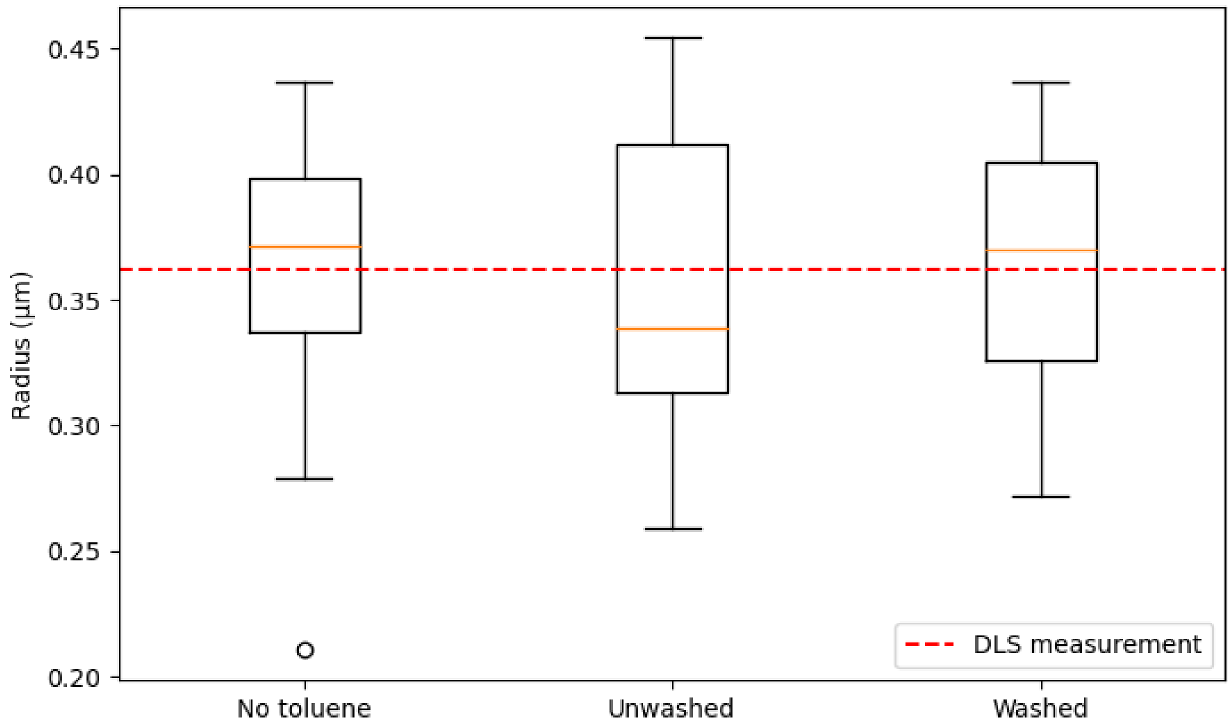

Figure S2: Box plot summarising manual measurements of colloid radii on the water-dodecane interfaces from cryo-SEM images. The orange lines correspond to the median values and the dotted line corresponds to colloid radii without toluene measured from DLS.

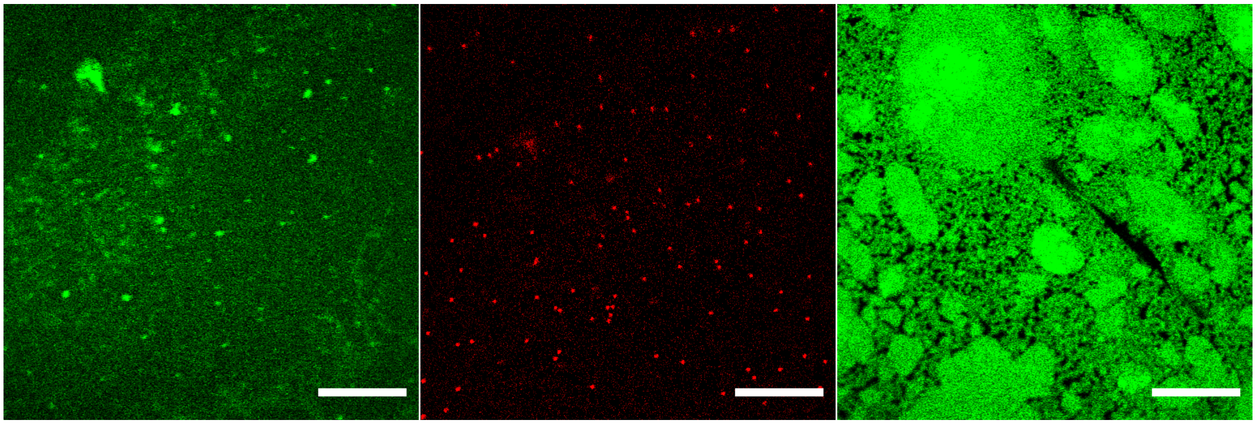

Figure S3: Left & Middle: Confocal microscopy of a homogenous interface in which the small NBD-dyed colloids (left) and large Nile Red dyed tracer colloids (middle) are excited respectively. Right: example of an inhomogenous interface that would not be used for analysis. Scale bar of 200μm
